# *Staphylococcus aureus *bacteriuria as a prognosticator for outcome of *Staphylococcus aureus *bacteremia: a case-control study

**DOI:** 10.1186/1471-2334-10-225

**Published:** 2010-07-29

**Authors:** Shingo Chihara, Kyle J Popovich, Robert A Weinstein, Bala Hota

**Affiliations:** 1 Department of Clinical Laboratory Medicine, Dokkyo Medical University, Tochigi, Japan; 2 Section of Infectious Diseases, Department of Medicine, Rush University Medical Center, 600 S Paulina, Suite 143 Academic Facility, Chicago, IL 60612, USA; 3 Division of Infectious Diseases, Department of Medicine, John H. Stroger Jr. Hospital of Cook County (CCH), Chicago, Illinois, USA

## Abstract

**Background:**

When *Staphylococcus aureus *is isolated in urine, it is thought to usually represent hematogenous spread. Because such spread might have special clinical significance, we evaluated predictors and outcomes of *S. aureus *bacteriuria among patients with *S. aureus *bacteremia.

**Methods:**

A case-control study was performed at John H. Stroger Jr. Hospital of Cook County among adult inpatients during January 2002-December 2006. Cases and controls had positive and negative urine cultures, respectively, for *S. aureus*, within 72 hours of positive blood culture for *S. aureus*. Controls were sampled randomly in a 1:4 ratio. Univariate and multivariable logistic regression analyses were done.

**Results:**

Overall, 59% of patients were African-American, 12% died, 56% of infections had community-onset infections, and 58% were infected with methicillin-susceptible *S. aureus *(MSSA). Among 61 cases and 247 controls, predictors of *S. aureus *bacteriuria on multivariate analysis were urological surgery (OR = 3.4, p = 0.06) and genitourinary infection (OR = 9.2, p = 0.002). Among patients who died, there were significantly more patients with bacteriuria than among patients who survived (39% vs. 17%; p = 0.002). In multiple Cox regression analysis, death risks in bacteremic patients were bacteriuria (hazard ratio 2.9, CI 1.4-5.9, p = 0.004), bladder catheter use (2.0, 1.0-4.0, p = 0.06), and Charlson score (1.1, 1.1-1.3, p = 0.02). Neither length of stay nor methicillin-resistant *Staphylococcus aureus *(MRSA) infection was a predictor of *S. aureus *bacteriuria or death.

**Conclusions:**

Among patients with *S. aureus *bacteremia, those with *S. aureus *bacteriuria had 3-fold higher mortality than those without bacteriuria, even after adjustment for comorbidities. Bacteriuria may identify patients with more severe bacteremia, who are at risk of worse outcomes.

## Background

Bacteriuria with *Staphylococcus aureus *is postulated to occur through a limited number of mechanisms--primarily ascending spread after instrumentation (e.g., urologic procedures or urethral catheterization) or hematogenous seeding of the genitourinary tract. The finding by Lee et al., that bacteremia is strongly associated with bacteriuria among patients infected with *S. aureus*, supports the notion that bacteremia is an important precursor for bacteriuria [1]. What is less clear, however, is whether in patients with *S. aureus *bacteremia, the finding of *S. aureus *in the urine holds any prognostic significance.

Among patients with *S. aureus *bacteremia, there are many prognostic factors, including host factors or comorbidities (age [2-10], Charlson score [2,11], immunosuppression [4], alcoholism [4], hemodialysis [4], acute renal failure [4], diabetes mellitus [8], recent hospitalization [11], mechanical ventilation [5], and acute severity of illness [12]); pathogen-specific factors (MRSA [3,13] superantigenic toxin production [14]); or characteristics of clinical presentation or management (meningitis [13] or altered mental status [11,12], community-acquired infection [4], severe sepsis or septic shock [3,4,6,7], lack of an infectious disease consult [4,15], prior antibiotic therapy [6], unknown [8] or persisting foci of infection [16], daily dose of penicillinase-stable penicillin < 4 grams [16], and inappropriate empiric treatment [6,12] or duration of treatment < 14 days [16]). The relation of occurrence of bacteriuria to outcome has been postulated [17], but the interactions with other risk factors warrant investigation. That bacteriuria might be a predictor of worse outcome in *S. aureus *bacteremia has biologic plausibility. For example, several innate defense mechanisms exist that prevent the development of urinary tract seeding in bacteremic patients: defensins, Toll-like receptor 4, and chemokine receptor CXCR1 [18,19], and these innate defenses may be overcome and the urinary tract seeded in the setting of higher bacterial loads [20].

Over the last 20 years, MRSA has emerged as an important cause of nosocomial bacteremia [21], and significant increases in the incidence of MRSA infections in community patients have been observed in the past few years [22-24]. Methicillin-resistance is a major risk for increased morbidity and mortality in *S. aureus *infection. Given the increasing burden of infection by community-associated MRSA (CA-MRSA) infections [22-24] and the suggestion that CA-MRSA strains may have greater virulence, predictors of morbidity and mortality are essential for allocation of clinical infections.

We hypothesized that for patients with *S. aureus *bacteremia, bacteriuria is an important marker of disease severity and predictor of worse outcomes. We examined this hypothesis by evaluating outcomes of *S. aureus *bacteremia in patients with and without bacteriuria. We also examined the impact of methicillin-resistance on outcomes among patients with *S. aureus *bacteremia with and without bacteriuria. Finally, using our cohort, we examined the predictors of bacteriuria.

## Methods

### Setting

A case-control study was performed for patients admitted to John H. Stroger Jr. Hospital of Cook County, a 464-bed inner-city safety-net hospital in Chicago, Illinois. The institutional review board reviewed the study and deemed it exempt from review.

### Study Design and Definitions

Our study included in-patients during January 2002 through December 2006. Cases were patients who had a positive urine culture for *S. aureus *within 72 hours of a positive blood culture for *S. aureus*. Controls were patients who had a negative urine culture for *S. aureus *within 72 hours of a positive blood culture *S. aureus*. Control patients were randomly sampled from all eligible patients with *S. aureus *bacteremia but without bacteriuria (i.e., with negative urine cultures), with the aim of 4 controls per case. Subsequently, chart reviews were performed and exclusion criteria were applied to sampled putative cases and controls to create a cohort for analysis.

Patients who were younger than age 18 years, who were seen only in the emergency department or in the clinic, or who did not have a urine culture performed within 72 hours of the positive blood cultures, were excluded. Only the first episode of *S. aureus *bacteremia per admission was assessed.

Using electronic data and chart review, we collected demographic and clinical data including mortality, ICU admission, length of stay, age, race, sex, Charlson score [25], comorbidities (HIV, diabetes mellitus, end stage renal disease requiring hemodialysis), alcoholism, intravenous drug use, presence of indwelling bladder catheter at the time of culture, previous urological procedure, duration of fever, duration of bloodstream infection, urinary symptoms, duration of chief complaint, organs and sites infected by *S. aureus *decided by the clinician, evidence of foreign material, susceptibilities of *S. aureus*, and time to appropriate antimicrobial therapy. For patients who died during their hospitalization, death was attributed to the bacteremia if so noted by the patient's discharge diagnosis or when there was a positive blood culture within 1 week preceding death.

*S. aureus *was deemed hospital-acquired if illness had onset > 72 hours after hospital admission. Cultures that turned positive within 72 hours of admission--community-onset--were divided into 2 groups: healthcare-associated infection was defined based on history of hospitalization, surgery, dialysis, or residence in a long-term care facility within 1 year or a presence of an indwelling catheter or percutaneous medical device (e.g., tracheostomy tube, gastrostomy tube, or bladder catheter) or a prior MRSA infection; patients with community-acquired infection did not have any of these risk factors [26].

### Statistical Methods

Categorical variables were examined with chi-square analysis and continuous variables were analyzed with the independent samples t test. For multivariable analysis, statistically important factors with p values <0.10 on univariate analysis were included in initial models, and Charlson score was included a priori based on the strong association between comorbidities and death, and our desire to appropriately adjust for comorbidities in our analysis. We used Cox proportional hazards regression analysis to assess the association of bacteriuria with time to *S. aureus *death or hospital discharge, adjusted for suspected effect modifiers as identified in univariate analysis. Explanatory variables were evaluated for adherence to the proportional hazards assumption. To assess the multivariable predictors of bacteriuria, logistic regression was used. Data analysis was performed using SPSS software (version 15.0, Chicago, IL).

## Results

During 5 years (January 1, 2002, through December 31, 2006), 67 patients with *S. aureus *bacteremia and bacteriuria were identified, and control sampling yielded 268 bacteremic, non - bacteriuric patients. Following chart reviews and application of exclusion criteria, there were 61 cases and 247 controls. Of all 308 episodes of bacteremia, 128 (41.6%) were due to MRSA.

The mean age of pooled dataset, created by joining case and control patients was 48.3 years; 68.5% were male, 181 (58.8%) were African-American, and 37 (12.0%) died during hospital admission. Sites involved by bacteremia were cardiac valves [31 (10.7%)], vascular catheters [59 (19.2%)], skin/soft tissue [57 (18.5%)], bone/joints [27 (8.8%)], lungs [41 (13.3%)], genitourinary tracts [22 (7.1%)], and other sites [11 (3.6%)]; for 106 (36.7%) patients, there was no documented organ involvement other than bloodstream.

**Figure 1:**
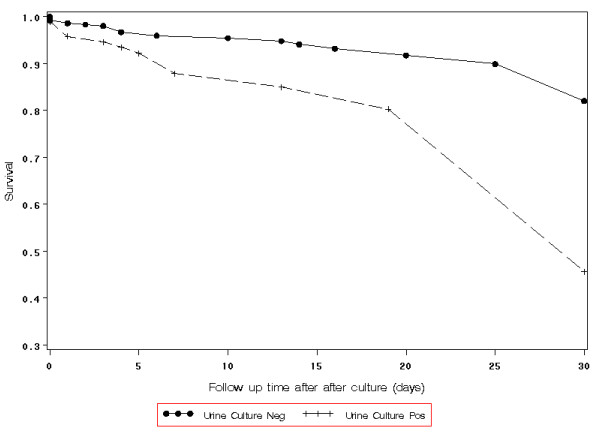
**Cox regression for survival analysis for *S. aureus *bacteremia patients with and without bacteriuria (p value = 0.004)**.

Hospital mortality rates were higher in *S. aureus *bacteremia patients with bacteriuria compared to patients without bacteriuria (39% vs 17%, respectively, p = 0.002) (table 1). In multiple Cox regression (table 2), significant predictors of death were positive urine culture (Hazard ratio 2.9; 95% CI, 1.4-5.9, p = 0.004), indwelling bladder catheter (2.0; 95% CI, 0.97-4.0, p = 0.06), and Charlson score (1.2; 95% CI, 1.1-1.3, p = 0.02); vascular catheter infection or phlebitis was associated with lower risk of death (0.27; 95% CI, 0.060-1.2, p = 0.09). Cox regression showed a significantly higher mortality rate in case than in control patients (figure 1). Neither methicillin-resistance nor the specific strain types of MRSA were predictors of mortality in univariate or multivariable analysis.

**Table 1:** Univariate analysis of predictors of death during admission among patients with *S. aureus *bacteremia and a contemporaneous urine culture obtained ^a^

Risk Factor^b^	Death during admission	No death during admission	OR (95% CI)	P value
N	36	253		

Demographics				

Male	22 (61)	176 (70)		0.31

Race/Ethnicity				0.46

Black	22 (61)	145 (50)		

Hispanic	9 (25)	44 (15)		

Other race	2 (6)	23 (8)		

White	3 (8)	41 (14)		

Age	51.6 (15.8)	47.6 (15.3)		0.15

Microbiological Factors				

Epidemiologic risk				0.76

Community associated	11 (31)	69 (27)		

Healthcare associated	8 (22)	71 (28)		

Hospital onset	17 (47)	113 (45)		

MRSA	17 (47)	103 (41)		0.46

Urine culture positive	14 (39)	43 (17)	3.11 (1.47 - 6.55)	0.002

Clinical Factors				

HIV	6 (17)	28 (11)		0.32

Bladder catheter	19 (53)	86 (34)	2.17 (1.07 - 4.39)	0.03

Charlson score	2.84	2.04		0.22

Hemodialysis	1 (3)	26 (11)	0.24 (0.03 - 1.90)	0.15

Immunosuppressed	8 (22)	45 (18)		0.52

Urological surgery	0 (0)	13 (5)		0.16

DM	13 (36)	70 (28)		0.30

IVDA	4 (11)	42 (17)		0.40

Prosthetic device	3 (8)	63 (25)	0.27 (0.08-0.92)	0.03

Alcoholism	7 (19)	40 (16)		0.58

ID consult or management^c^	18 (50)	128 (60)		0.25

Line/phlebitis	2 (6)	56 (22)	0.21 (0.05-0.89)	0.02

Skin soft tissue infection	7 (19)	50 (20)		0.96

Bone/joint infection	2 (6)	25 (25)		0.40

Pulmonary infection	6 (17)	35 (14)		0.65

GU infection	0 (0)	22 (9)		0.05

Incubation Period	26.8 (67.4)	12.4 (24.0)		0.21

Outcome				

Fever days	2.7 (4.8)	3.7 (5.0)		0.11

Time to effective therapy	1.06 (2.6) days	1.8 (3.7) days		0.12

Length of stay after culture	10.1 (11.3) days	13.5 (14.1) days		0.17

Footnotes: ^a ^Contemporaneous urine cultures are performed within 72 hours of the blood culture. ^b^Data represent count (%) and mean (standard deviation) for count and continuous data, respectively. ^c^Patient management was either primarily by infectious diseases clinician or with infectious diseases consultation.

**Table 2:** Multiple Cox regression analysis of factors predictive of death among patients with *S. aureus *bacteremia with one contemporaneous urine culture obtained

Risk factor	Hazard Ratio (95% CI)	P value
Urine culture positive for *S. aureus*	2.87 (1.411-5.851)	0.004

Bladder catheter present	1.96 (0.966-3.965)	0.063

Line infection/phlebitis	0.268 (0.060-1.199)	0.085

Charlson score	1.183 (1.063-1.316)	0.018

More positive urine cultures were seen in community-onset (both community-acquired and healthcare-associated) compared to hospital-acquired bacteremia (46% vs 23%, respectively, p < 0.001); in HIV-positive patients compared to HIV-negative patients (19% vs 10%, p = 0.05); and in patients with recent urological surgery (11% vs 3%, p = 0.03) or genitourinary infection (21% vs 4%, p < 0.001) compared with those without genitourinary problems (table 3). In multivariable analysis (table 4), urological surgery (OR 4.5, p = 0.045) and genitourinary infection (OR 5.7, p = 0.002) were significantly associated with positive urine cultures.

**Table 3:** Univariate analysis of risk factors for bacteriuria among patients with *S. aureus *bacteremia

Risk Factor^a^	Urine culture positive	Urine culture negative	OR (95% CI)	P value
N	57	232		

Demographics				

Age	49.1 (13.8)	47.9 (13.8)		0.59

Male	40 (70)	158 (68)		0.76

Race/Ethnicity				0.10

Black	26 (46)	141 (61)		

Hispanic	14 (25)	39 (17)		

Other Race	4 (7)	21 (9)		

White	13 (22)	31 (13)		

Microbiological factor				

Epidemiological Risk				

Community onset	26 (46)	54 (23)		<0.001

Healthcare associated	18 (32)	61 (26)		

Hospital onset	13 (23)	117 (50)		

MRSA	21 (37)	99(43)		0.42

Clinical Factors				

Immunosuppressed	9 (16)	44 (19)		0.58

Incubation Period	15.7 (30.5)	13.8 (33.4)		0.68

Charlson Score	2.05	2.15		0.801

Hemodialysis	3 (5)	24 (10)		0.24

HIV	11 (19)	23 (10)	2.17 (1.00 - 4.77)	0.05

Endocarditis	7 (12)	17 (7)		0.22

Bladder catheter	20 (35)	85 (37)		0.83

Urological surgery	6 (11)	7 (3)	3.78 (1.22 - 11.73)	0.03

DM	19 (33)	64 (28)		0.39

IVDA	7 (12)	39 (17)		0.40

Prosthetic device	11 (19)	55 (24)		0.48

Alcoholism	11 (19)	36 (16)		0.49

Line infection/phlebitis	6 (11)	52 (22)	0.41 (0.17 - 1.00)	0.05

Skin soft tissue infection	10 (18)	47 (20)		0.64

Bone/joint infection	7 (12)	20 (9)		0.39

Pulmonary infection	12 (21)	29 (13)	1.87 (0.89 - 3.94)	0.10

GU infection	12 (21)	10 (4)	5.92 (2.41 - 14.54)	<0.001

Footnotes: ^a^Data represent count (%) and mean(standard deviation) for count and continuous data, respectively.

**Table 4:** Multiple Cox regression analysis of risk factors for bacteriuria in patients with *S. aureus *bacteremia

Risk factor	OR (95% CI)	P value
Urological surgery	4.47 (1.03-19.30)	0.045

GU infection	5.70 (1.90-17.12)	0.002

Factors included in Backward LR analysis: location of acquired infection (community-acquired vs hospital-acquired vs healthcare-associated); race; urological surgery; presence of lung infection; presence of genitourinary tract infection; duration of bloodstream infection

## Discussion

In our case-control study, patients who had *S. aureus *bacteremia and a positive urine culture for *S. aureus *had significantly higher mortality than did bacteremic patients with a negative urine culture. This was true even after adjustment for multiple covariates including use of bladder catheters, presence or absence of lower urinary tract symptoms, or recent urological surgeries, which are known risk factors for a positive urine culture, significant factor in the multivariable analysis such as presence of line infection or phlebitis or presence of comorbidities as measured by the Charlson score [2,11]. The conclusion that *S. aureus *bacteriuria patients have worse prognosis compared to patients without *S. aureus *bacteriuria among *S. aureus *bacteremia patients may be useful to clinicians when deciding whether to monitor a patient on the ward or the ICU and when explaining the prognosis of the illness to the patient and his family.

*S. aureus *is a common pathogen in the community and in hospitals. *S aureus *causes significant mortality and morbidity but is an infrequent cause of urinary tract infection [27]. In patients with *S. aureus *bacteremia, a positive urine culture is typically attributed to ascending infection or to hematogenous spread. Predictors of a positive urine culture for *S. aureus *include presence of indwelling bladder catheters, urinary tract obstruction, instrumentation, or surgery [27,28]. We hypothesized that *S. aureus *appears in the urine in bacteremic patients because of higher burden of organisms and thus may portend worse outcome.

A recent study examining the relationship of bacteriuria with mortality found a two-fold increased risk of death in patients with concomitant *S. aureus *bacteriuria and bacteremia [17]. The current study adds to published literature by including significant numbers of methicillin-resistant isolates and by using statistical methods appropriate for varying time at risk. The association of bacteriuria with increased risk of mortality even after adjustment for comorbidities was similar in both studies, suggesting that bacteriuria provides useful prognostic information in the setting of *S. aureus *bacteremia. In addition, based on our findings, it appears that this is an effect independent of methicillin-resistance and that secular trends in incidence of MRSA are unlikely to alter this association.

There are several limitations to our study. The patients in the study were cared for at a single center and it may be difficult to generalize our findings to other hospitals. The Cook County Bureau of Health Services is the largest provider of indigent care in Cook County and has seen large increases in rates of community-associated *S. aureus *infection, and our estimates likely represent robust measures of risk for mortality. A second limitation is that this was a retrospective study and depended on documentation in the chart, which may have resulted in misclassification errors in assessing comorbidities and other covariates. Non-differential misclassification tends to bias towards the null, which suggests that our estimates of risk are likely conservative. In addition, electronic data were used in this study and were prospectively collected at the time of care; these data were not subject to problems inherent in reviews of written documentation Finally, in our study, we had an overall mortality rate of 12% which was at the lower end of published mortality rates--12-46% [3-13,27,29,30]--in this setting, suggesting that our cohort may have had fewer comorbidities and lower attributable mortality that in other published data. This may be a result of the significant burden of CA-MRSA in our patient population.

## Conclusions

In patients with *S. aureus *bacteremia, a positive urine culture for *S. aureus *may be used to identify patients at higher risk of death. Patients who have *S. aureus *bacteremia and more comorbidities are at increased risk for higher mortality rate.

## Competing interests

Bala Hota is a member of the speaker's bureau for Pfizer. All other authors have no competing interests.

## Authors' contributions

SC participated in the design of the study, performed the chart reviews, participated in some statistical analysis and drafted the manuscript. KJP participated in obtaining the data for the study and proofreading the manuscript. RAW participated in the design of the study and proofreading the manuscript. BH participated in the design of the study, performed statistical analysis and proofread the manuscript. All authors read and approved the final manuscript.

## Pre-publication history

The pre-publication history for this paper can be accessed here:

http://www.biomedcentral.com/1471-2334/10/225/prepub
